# Computational Analysis on the Performance of Elongated Liquid Crystal Biosensors

**DOI:** 10.3390/mi14101831

**Published:** 2023-09-26

**Authors:** Reza Shadkami, Philip K. Chan

**Affiliations:** Department of Chemical Engineering, Toronto Metropolitan University, 350 Victoria Street, Toronto, ON M5B 2K3, Canada; reza.shadkami@torontomu.ca

**Keywords:** liquid crystal biosensors, anisotropic, droplet, director reorientation, elongated

## Abstract

Elongated ellipsoidal liquid crystal microdroplet reorientation dynamics are discussed in this paper for biosensor applications. To investigate the effect of elongated droplets on nematic liquid crystal droplet biosensors, we simulated a model of a liquid crystal droplet using ellipse geometry. Director reorientation is examined in relation to the elongated droplet shape. In addition, we examined aspect ratio as a factor affecting biosensor response time in relation to surface viscosity and anchoring energy. Finally, the findings suggest that the aspect ratio should be taken into account when designing biosensors. These results can be used to develop more effective biosensors for a variety of applications. This model then predicts the director reorientation angle, which is dependent on the anchoring energy and surface viscosity. This model further suggests that both surface viscosity and homeotropic anchoring energy play an important role when it comes to the director reorientation angle. We developed and applied a nonlinear unsteady-state mathematical model utilizing torque balance and Frank free energy according to the Leslie–Ericksen continuum theory for simulating elongated nematic liquid crystal biosensor droplets with aqueous interfaces. Using the Euler–Lagrange equation, a transient liquid crystal–aqueous interface realignment is modeled by changing the easy axis when surfactant molecules are added to the interface. The realignment at the surface of the droplet is assumed to be driven by the effect of the surfactant, which causes an anchoring transition. According to the results, the response time of the biosensor depends on the aspect ratio. Therefore, the elongation has the potential to control biosensing response time. The result of our study provides a better understanding of director reorientation in elongated liquid crystal droplets in biosensing applications through the numerical results which are presented in this paper.

## 1. Introduction

Biosensors combine receptors and transducers to detect biological and chemical analytes. In a biosensor, the receptor binds to the chemical agent, while the transducer converts this interaction into an optical signal that can be amplified to specify biomolecule recognition. The biosensor produces a signal proportional to the concentration of analyte in the sample. Liquid crystals can be used for sensing applications ranging from environmental pollution detection and medical diagnostics to monitoring biological processes. Because of the optical properties of liquid crystals, their long-range orientational order and their sensitivity to interface interactions make them effective for detecting and responding to biomolecules [1,2,3].

In general, liquid crystal biosensors are categorized as thermotropic and lyotropic. A lyotropic liquid crystal is water-based and dissolves in water. During biosensor applications, aqueous solution and target samples can be directly added to liquid crystals. On the other hand, thermotropic liquid crystals are immiscible to water and form an interface with water. However, in thermotropic liquid crystal biosensing, thermotropic liquid crystals act as a substrate in aqueous phase contact with lyotropic liquid crystals [4,5]. In this paper, we investigated thermotropic liquid crystal biosensors with aqueous interfaces; liquid crystal refers to thermotropic liquid crystal wherever it is used.

Several past experimental results show that liquid crystals dispersed in water are capable of sensing viruses and bacteria. Researchers reported that the chemical analytes adsorb to the surface of the liquid crystal droplet and trigger a change in the molecular alignment at the interface, thus changing the orientation of the director field within the droplet from bipolar to radial. Also, that shows that liquid crystal droplets dispersed in the water exhibit homeotropic ordering at the surface when molecules of amphiphilic materials are adsorbed. These results show that configuration within liquid crystal droplets highly depends on adsorption. There are also many other factors that impact the orientation of droplets, such as temperature, shape of geometry, surface property, and external fields [6,7,8,9,10,11].

In this study, we focused on two types of optical textures that have been observed in experimental studies. First, the bright optical appearance of liquid crystals in water in the absence of biomolecules and any external fields. Second, the dark cross texture caused by the presence of biomolecules that is induced by the homeotropic alignment of liquid crystal molecules at the interface [6,12,13,14]. 

**Figure 1:**
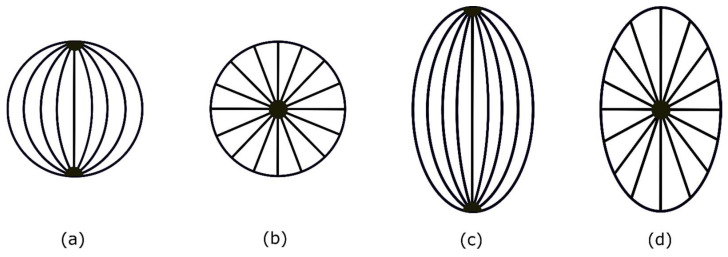
(**a**) Bipolar configuration of liquid crystal droplets where liquid crystal molecules are tangential and planar to the surface and two opposed point defects appear diametrically. (**b**) The radial configuration of a liquid crystal droplet with a single defect point at the center of the droplet. (**c**) The elongated bipolar configuration; (**d**) elongated radial configuration.

Director configuration within a droplet is determined via the minimizing energy of the bulk and surface. A liquid crystal droplet in pure water forms a tangential orientation under the planar anchoring condition. A parallel alignment of the director, in which it is aligned tangentially to the droplet interface, results in a bipolar configuration in the absence of surfactants or any other external fields. Bipolar configurations have diametrically opposed defect points at the poles of the droplet, as shown in Figure 1.

Researchers have developed a method for synthesizing anisometric microdroplet emulsions with anisotropic optical properties, which can be applied to control liquid crystal droplet configurations as well as surfactant adsorption at liquid crystal aqueous interfaces. This method could be used in a variety of applications, such as in optical devices. They prepared different shapes of liquid crystals and studied their internal configuration within droplets. According to the findings, this method could be used to create optically functional microdroplet emulsions for a wide range of applications. It was found that droplet shape had a crucial role to play in controlling droplet configurations in the presence of the surfactant [1,15,16]. 

This model is a modification of the mathematical model that we used for liquid crystal droplets in our previous project [17]. An elongated nematic liquid crystal is modeled to simulate the transient configuration of the director between bipolar and radial when a chemical analyte adsorbs on its surface. The model can be used to understand how the system responds to different elongated droplet shapes. Moreover, the model can be used to investigate how droplet elongated shape, anchoring energy, and surface viscosity influence director configuration. Using an ellipse with a defined aspect ratio, we investigated the effects of droplet shape by changing the aspect ratio of the ellipse. When the aspect ratio approaches unity, the ellipse becomes a circle equivalent to a spherical droplet. 

We used average angle and characteristic time as performance criteria for evaluating the performance of a biosensor to study the system response time and orientation dynamics within elongated droplets. Mathematical modeling and computer simulation have not been explored in the application of biosensors for elongated liquid crystal droplets, as per our knowledge.

For our study, we assumed that a sufficient surfactant concentration results in a sudden transition from planar to homeotropic of the easy axis [18]. Our study does not consider the liquid crystal response to biomolecules upon interaction with topological defects, as reported by Lin et al. [19], and it is beyond the scope of our current work. 

Since the configuration of liquid crystal droplet emulsion depends on droplet size, we limited our study of elongated liquid crystal droplets to the case where the cross-section area of spherical droplets and elongated droplets are the same, as well as the volume [20,21]. 

It was also assumed that the ellipsoidal liquid crystal droplets with bipolar configurations formed under homogeneous tangential anchoring and radial structure formed under homogeneous homeotropic anchoring. Also, it was assumed that by increasing the homeotropic anchoring strength of the liquid crystal at the interface, the surface alignment is subjected to the transition from planar anchoring to homeotropic anchoring under the influence of analytes [22].

Furthermore, in this study, we assumed that adsorption-driven perpendicular anchoring was responsible for obtaining radial configurations of liquid crystal droplets that were caused by biomolecular adsorption, in addition to constant temperature and uniform interface properties [22,23]. Moreover, this study assumed the defect changed abruptly. We did not consider other possible configurations of liquid crystal droplets caused by defect migration. 

As a result of forming the radial configuration, the dark crossed optical pattern was observed experimentally. The dark-crossed optical pattern of the liquid crystal droplets is the result of the radial configuration and the adsorption of biomolecules. The concentration of biomolecules can affect the intensity of the optical response, with higher concentrations resulting in stronger optical responses.

This dark-crossed texture results from the uniaxial nematic phase experiencing two different refractive indices when light propagates through liquid crystal droplet films. Thus, the optical appearance of liquid crystal emulsions was altered by transitions in the orientation of droplets from bipolar to radial, which was induced by the adsorption of biomolecules. Therefore, these changes in optical textures correspond to changes in director field orientation. An optical pattern can be investigated by studying the dynamics of reorientation. Thus, droplet orientation can be used as an indicator of liquid crystal biosensor performance [6,7,24,25]. 

## 2. Modeling and Computational Method

The governing equation was formulated in accordance with Leslie–Ericksen theory by applying torque balance incorporated with Frank energy. The distortion free energy density of nematic liquid crystals was calculated using Frank continuum theory and expressed as follows [26,27]:

(1) $$F_{d}=\frac{1}{2}K_{11}{\left(\nabla \cdot n\right)}^{2}+\frac{1}{2}K_{22}{\left(n\cdot \left(\nabla \times n\right)\right)}^{2}+\frac{1}{2}K_{33}{\left|n\times \left(\nabla \times n\right)\right|}^{2}$$

The coefficients *K*_11_, *K*_22_, and *K*_33_ are the splay, twist, and bend elastic constants, respectively.

A system of liquid crystal droplets undergoes a reorientation or disturbance from its equilibrium state due to these three possible types of disturbances in nematic liquid crystal. Typically, these constants are in the range of 10^−11^ N, which indicates how stiff the liquid crystal system is with respect to distortions.

To determine the governing equation for reorienting the director within a droplet, a balance of torques is applied to the director field as a continuum medium. The torque balance is written in cartesian vector notation as follows:

(2) $$\;\Gamma_{e}+\Gamma_{v}=0$$

where the two terms are **Γ**_v_, viscous torque, and **Γ**_e_, elastic torque. The constitutive equations to calculate elastic and viscous torque can be calculated by using Equations (3) and (4):

(3a) $$\Gamma_{e}=n\times h$$

where **h** is the molecular field that is expressed with the following constitutive equations:

(3b) $$h=h_{S}+h_{T}+h_{B}$$

(3c) $$\;h_{S}=K_{11}\nabla \left(\nabla \cdot n\right)$$

(3d) $$h_{T}=-K_{22}\left\{a\nabla \times n+\nabla \times \left(an\right)\right\}$$

(3e) $$\;h_{B}=K_{33}\left\{b\times \nabla \times n+\nabla \times \left(n\times b\right)\right\}$$

where *a* and **b** are defined by the following constitutive equations:

(3f) a=n×∇×n

(3g) b=n×∇×n

The viscous torque can be calculated by using Equation (4a):

(4a) $$\;\Gamma_{v}=-n\times \left(\gamma_{1}N+\gamma_{2}A\cdot n\right)$$

where $\gamma_{1}$ and $\gamma_{2}$ are the rotational and irrotational torque coefficients. The rate of deformation tensor **A** and vorticity tensor **Ω** is defined as follows:

(4b) $$\;A=\frac{1}{2}\left[{\left(\nabla V\right)}^{T}+\nabla V\right]$$

(4c) $$\;\Omega =\frac{1}{2}\left[{\left(\nabla V\right)}^{T}-\nabla V\right]$$

where **V** is the velocity and **N** is defined as the angular velocity of the director relative to that of the fluid, which is defined in Equation (5) below. It is assumed that the liquid crystal is stationary within the droplet, and there is no bulk movement. As a result, **A** and **Ω** are zero.

(5) $$\;N=\dot{n}-\Omega \cdot n\;$$

where $\dot{n}$ represents material time derivative. 

### 2.1. Mathematical Model

**Figure 2:**
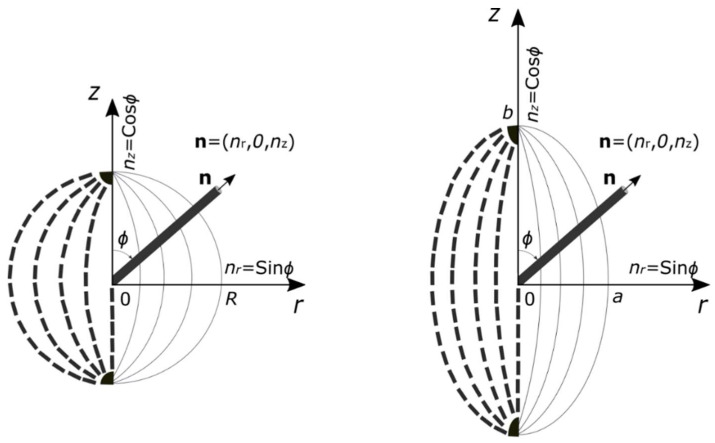
The schematic illustrates a two-dimensional bipolar droplet and an elongated droplet in a cylindrical coordinate system. The *z*-axis is along the bipolar droplet axis of symmetry. The ellipse represents an elongated droplet with *a* and *b* as the minor and major lengths, respectively. *R* represents the radius of the spherical droplet. **n** is the director and *ϕ* is the polar angle, which provides the angle relative to the *z*-axis in the *r*–*z* plane.

The governing equations and Frank energy are derived by considering the spherical droplet cross-section as an ellipse. Figure 2 shows cross-sections of a spherical and an elongated bipolar droplet defined in the cylindrical coordinate system (*r*, *θ*, *z*). The transient two-dimensional director field **n** is described as follows:

(6) $$\;n\left(r,z,t\right)=\left(\sin\phi \left(r,z,t\right),\;0,\;\cos\phi \left(r,z,t\right)\right)$$

where ϕ is the polar angle measured from the *z*-axis, which is space- and time-dependent. We considered this study to be a two-dimensional investigation of director reorientation dynamics within an ellipse since bipolar droplets are axisymmetric. A spherical droplet is described by a circle with radius *R* and areas *A*_c_ = π*R*^2^. An elongated droplet is described by an ellipse with a minor axis a, major axis *b*, and area *A*_e_ = π*ab*. Our study assumed that spherical droplets and elongated droplets have the same volume and equal cross-section areas. Furthermore, the aspect ratio is defined as *c* = *a*/*b* for the ellipse [20,28]. The aspect ratio was used to analyze the effects of ellipse shapes on liquid crystal droplet reorientation dynamics. 

The Frank energy can be expressed in a two-dimensional cylindrical coordinate system by substituting Equation (6) into Equation (1):

(7) $$\begin{array}{ll} F_{d}=\frac{1}{2}\frac{K_{11}\sin^{2}\phi }{r} & +\frac{1}{2}K_{11}r{\left(\frac{\partial \phi }{\partial z}\right)}^{2}\sin^{2}\phi -K_{11}\frac{\partial \phi }{\partial z}\sin^{2}\phi +\frac{1}{2}K_{11}\frac{\partial \phi }{\partial r}\sin\left(2\phi \right)-\frac{1}{2}K_{11}r\frac{\partial \phi }{\partial z}\frac{\partial \phi }{\partial r}\sin\left(2\phi \right)\\ & +\frac{1}{2}K_{11}r{\left(\frac{\partial \phi }{\partial r}\right)}^{2}\cos^{2}\phi +\frac{1}{2}K_{33}r{\left(\frac{\partial \phi }{\partial r}\right)}^{2}\sin^{2}\phi +\frac{1}{2}K_{33}r\frac{\partial \phi }{\partial z}\frac{\partial \phi }{\partial r}\sin\left(2\phi \right)+\frac{1}{2}K_{33}r{\left(\frac{\partial \phi }{\partial z}\right)}^{2}\cos^{2}\phi \end{array}$$

In the absence of an external field, the director configuration of a droplet is determined using the balance between bulk and surface energy. The total free energy of liquid crystals within a droplet can be expressed as follows:

(8) $$F=\underset{V}{\int }F_{d}dV+\underset{S}{\int }F_{s}ds$$

where *F*_d_ can be calculated from Frank free energy (1), and *F*_s_ can be determined by using the Rapini-Papoular expression as follows [29]:

(9) $$\;F_{s}=\frac{1}{2}W_{\phi }\sin^{2}(\phi_{s}-\phi_{e})\;$$

The anchoring coefficient $W_{\phi }$ describes the strength of surface anchoring. Droplet anchoring angles $\phi_{s}$ and $\phi_{e}$ are the actual and preferred anchoring angles at droplet surfaces. The director aligns with the easy axis when there is strong anchoring at the surface. In contrast, weak anchoring allows the system to evolve towards an intermediate equilibrium by balancing viscous and elastic torques, depending on the strength of anchoring.

The Euler–Lagrange equation describes the distortion of a liquid crystal droplet’s surface through the balance of generalized and frictional forces. The calculus of variations was used to derive the equation for the dynamics of thermotropic liquid crystal droplets at the interface with aqueous liquids. The system consists of one generalized coordinate, ϕ. For our system, the Rayleigh generalized dissipation function was simplified to drive the Euler–Lagrange equation as follows [30]:

(10) $$\;\frac{\partial R^{s}}{\partial \dot{\phi }}+\Phi_{\phi }=0$$

where $R^{s}$ is the Rayleigh dissipation function for surfaces, $\;\Phi_{\phi }$ is denoted as elastic forces, and ϕ represents a generalized coordinate. Then, the Euler–Lagrange equation is as follows:

(11) $$\;\frac{\partial R^{s}}{\partial \dot{\phi }}=\lambda^{s}{}^{*}\frac{\partial \phi }{\partial t}$$

where the surface viscosity governing the surface director orientation at the liquid crystal droplet aqueous interface is indicated by *λ*^s*^ and an overdot on the function indicates time differentiation. 

System energy consists of bulk and surface components. The contribution of surface realignment and bulk to the variation of the total free energy of the system is found by considering Equations (7), (9) and (10), as below:

(12) $$\;F=\underset{V}{\int }F_{d}\left(\phi ,\;\nabla \phi \right)dV+\underset{S}{\int }F_{s}\left(\phi \right)ds$$

The variation of *F* can be written as below:

(13) $$\;\delta F=\underset{V}{\int }\left(\frac{\partial F_{d}}{\partial \phi }\delta \phi +\frac{\partial F_{d}}{\partial \nabla \phi }\delta \nabla \phi \right)dV+\int_{S}\left(\frac{\partial F_{s}}{\partial \phi }\delta \phi \right)dS$$

By applying the divergence theorem, the equation is as follows:

(14) $$\;\delta F=\underset{V}{\int }\left(\frac{\partial F_{d}}{\partial \phi }\delta \phi -\nabla \frac{\partial F_{d}}{\partial \nabla \phi }\delta \nabla \phi \right)dV+\int_{S}\left(\frac{\partial F_{d}}{\partial \phi }\delta \phi \cdot \nu +\frac{\partial F_{s}}{\partial \phi }\delta \phi \right)dS$$

In this equation, ***ν*** is the unit normal vector directed outward from the enclosing surface *S* of volume *V*. Then, the elastic forces at the surface can be expressed as follows:

(15) $$\;\Phi_{\phi }=\frac{\partial F_{d}}{\partial \nabla \phi }\nu +\frac{\partial F_{s}}{\partial \phi }$$

### 2.2. Governing Equation and Auxiliary Conditions

The scaling relations were used to nondimensionalize the governing equations and boundary conditions as follows:

(16a) $$\;K_{ii}{}^{*}=\frac{K_{ii}}{K},\;\left(\mathrm{for}\;i=1,\;3\right)$$

(16b) $$K=\frac{1}{2}\left(K_{11}+K_{33}\right)$$

(17) $$\;r^{*}=\frac{r}{R}$$

(18) $$\;z^{*}=\frac{z}{R}$$

(19) $$\;a^{*}=\frac{a}{R}$$

(20) $$\;b^{*}=\frac{b}{R}$$

(21) $$\;t^{*}=\frac{tK}{\gamma_{1}R^{2}}$$

In these relations, superscript asterisks denote dimensionless variables. 

The non-dimensional parameters of surface viscosity and anchoring energy are defined as follows:

(22) $$W=\frac{1}{2}\frac{RW_{o}}{K}$$

(23) $$\;\lambda^{s}{}^{*}=\frac{2\lambda^{s}}{\gamma_{1}R}$$

The governing equation that describes the behavior of the orientation within the elongated droplet was derived by incorporating Equations (2)–(6) and applying scaling Equations (16)–(21). Therefore, a two-dimensional, nonlinear partial differential equation that is time-dependent is as follows:

(24) $$\frac{\partial \phi }{\partial t^{*}}=\kappa_{1}\frac{\partial^{2}\phi }{\partial r^{*}{}^{2}}+\kappa_{2}\frac{\partial^{2}\phi }{\partial z^{*}{}^{2}}+\kappa_{3}\frac{\partial^{2}\phi }{\partial r^{*}\;\partial z^{*}}+\kappa_{4}{\left(\frac{\partial \phi }{\partial r^{*}}\right)}^{2}+\kappa_{5}{\left(\frac{\partial \phi }{\partial z^{*}}\right)}^{2}+\kappa_{6}\frac{\partial \phi }{\partial r^{*}}\frac{\partial \phi }{\partial z^{*}}+\kappa_{7}\frac{\partial \phi }{\partial r^{*}}+\kappa_{8}\frac{\partial \phi }{\partial z^{*}}+\kappa_{9}$$

where the spatially and angle-dependent elastic functions {*κ_i_*}, *i* = 1, 2, …9 are provided in Appendix A.

The following first-order time-dependent partial differential equations were obtained by applying Equations (7)–(15), the scaling relations (16)–(21), and Equation (24). Hence, the Euler–Lagrange equation describes liquid crystal motion at an interface as follows:

(25) $$\;\lambda^{s*}\frac{\partial \phi }{\partial t^{*}}=\kappa_{10}+\kappa_{11}\frac{\partial \phi }{\partial r^{*}}+\kappa_{12}\frac{\partial \phi }{\partial z^{*}}+W_{\phi }\sin\;\left[2\left(\phi -\phi_{e}\right)\right]$$

By considering *A*_c_ = *A*_e_, dimensionless lengths a* and b* are related to aspect ratio *c* and are defined as follows [26]:

(26a) $$a^{*}=\frac{1}{\sqrt{c}}$$

(26b) $$b^{*}=\sqrt{c}$$

As shown in Figure 2, the two-dimensional droplet has symmetry about the *z*-axis, so the governing Equations (24) and (25) can be solved numerically for half of a circle where r* > 0. Then, the initial and boundary conditions are defined as follows:
$\phi =\phi_{i}\left(r^{*},z^{*},t^{*}\right)$$\mathrm{at}\;t^{*}=0,$$r^{*}\geq 0,$$-1\;\leq z^{*}\leq 1$(27)$\frac{\partial \phi }{\partial r^{*}}=0$$\mathrm{at}\;t^{*}\geq 0,$$r^{*}=0,$$-1\;\leq z^{*}\leq 1$(28)$\lambda^{s*}\frac{\partial \phi }{\partial t^{*}}=\kappa_{10}+\kappa_{11}\frac{\partial \phi }{\partial r^{*}}+\kappa_{12}\frac{\partial \phi }{\partial z^{*}}+W_{\phi }\sin\;\left[2\left(\phi -\phi_{e}\right)\right]$(29)
$\mathrm{at}\;t^{*}\geq 0,$$r^{*}>0,$$z^{*}={\left(b^{*}{}^{2}-c^{2}r^{*}{}^{2}\right)}^{1/2}$

### 2.3. Numerical Method of Solution

Following spatial discretization, a set of nonlinear ordinary time-dependent differential equations are obtained, which are simultaneously solved using a Newton–Raphson iteration scheme. Our mathematical model was developed using Mathematica software v. 12.2 [31]. For the numerical solution, the Galerkin finite element method with the bilinear basis function was used. Convergence is assumed when the difference between the two vectors of sequence solutions is less than 10^−6^. The first-order forward finite difference method is used to discretize time, and the implicit Euler predictor-corrector method is used to integrate time.

Our mathematical model consists of nonlinear unsteady partial differential equations governed by transient boundary conditions. The mesh was generated with rectangular elements that discretize the two-dimensional droplet geometry. In addition, the initial dimensionless time step considered was 10^−5^ [32,33,34,35].

We developed the finite element program using FORTRAN 95 as the programming language [36,37] and compiled it using the Intel compiler. LAPACK solver routines from the Intel Math Kernel Library [38] were used to solve linear algebraic equations. The analysis and visualization of our results followed the same procedure we had followed previously [39]. Our analysis was performed on the Digital Research Alliance of Canada’s Cedar and Graham clusters using high-performance computing resources.

## 3. Result and Discussion

This section presents selective numerical results obtained from the simulation study which meet the objectives specified in studies. A mean magnitude of orientation angle was defined [20,39] as an indicator of orientation dynamics. Since the magnitude angle is related to the optical texture and transmittance, it is used to investigate the influence of droplet shape and surface properties on liquid crystal droplet biosensor performance.

The mean magnitude of the orientation angle 〈‖ϕ‖〉 within the elongated droplet is defined as follows:

(30) $$\phi =\frac{1}{\pi }\int_{-b^{*}}^{b^{*}}\int_{-a^{*}}^{a^{*}}\left|\phi \right|dr^{*}dz^{*}$$

In this study, the effect of droplet size was not studied, and it was assumed the radial configuration was a result of homotropic anchoring on the surface of the liquid crystal droplet caused by the surfactant.

**Figure 3:**
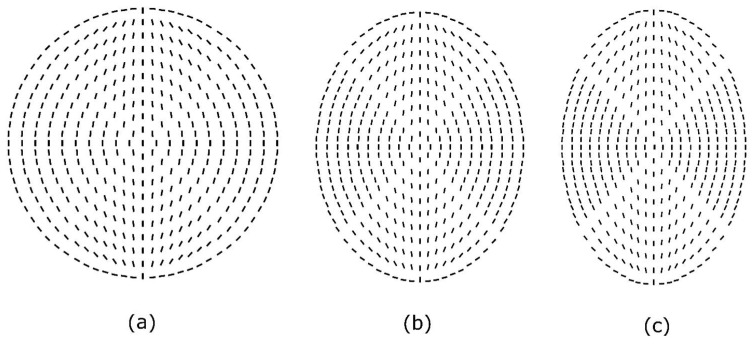
The figure illustrates the steady-state director configuration of a bipolar nematic droplet with various aspect ratios as the initial conditions. (**a**) *c* = 1.0, (**b**) *c* = 1.3, and (**c**) *c* = 1.5, in which *c* is aspect ratio. Aqueous solutions cause tangential anchoring at the surface of droplets and lead to bipolar orientation within the droplet.

For different aspect ratios, Figure 3 illustrates the director configuration of a bipolar nematic droplet based on the model by Xu et al. [40]. The configurations described here are the steady-state initial conditions used in the simulation related to that aspect ratio. Because of mirror symmetry about the *rz* plane, the simulation was performed in two dimensions. By utilizing that, the computational time is greatly reduced.

In this study, a number of case studies were conducted to investigate the aspect ratio range within 1.1 ≤ *c* ≤ 1.8. We examined the process of transition from bipolar to radial within an elongated droplet in relation to dimensionless homeotropic anchoring energy and dimensionless surface viscosity.

**Figure 4:**
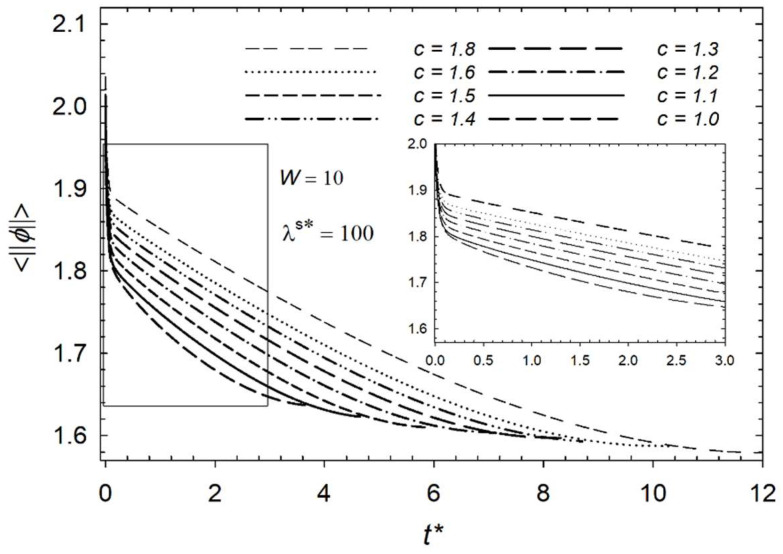
This figure illustrates how the mean magnitude of the orientation angle changes through dimensionless time t* with respect to aspect ratio *c* with surface parameters *λ*^s^* = 100 and *W* = 10.

**Figure 5:**
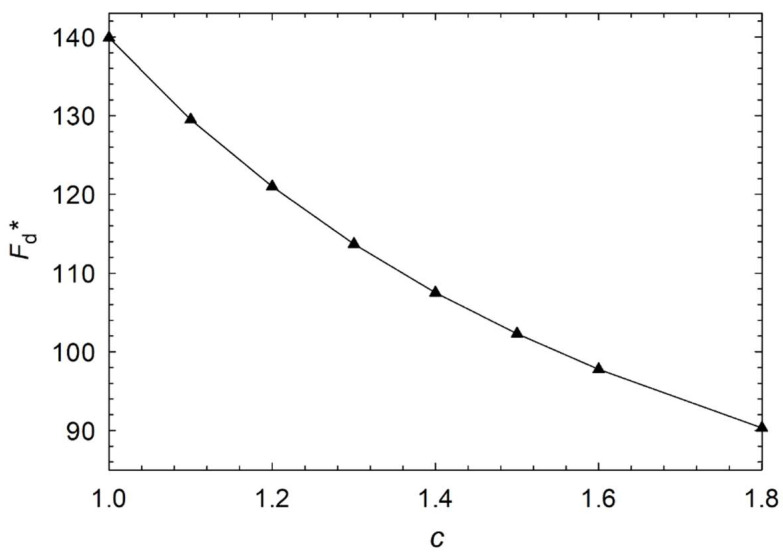
Total dimensionless stored distortion free energy versus aspect ratio in the steady-state condition for the bipolar configuration.

First, we compared the mean magnitude angle <||ϕ||> with regard to aspect ratios as the process proceeds, as shown in Figure 4. In this case, we studied dimensionless homeotropic anchoring energy *W* = 10 and the dimensionless surface viscosity *λ*^s^* = 100. As expected, the mean magnitude of the angle decreases with time. However, a smaller aspect ratio indicates a faster transition from a bipolar orientation to a radial orientation. This is due to the fact that with a smaller aspect ratio, the higher distortion elastic energy in droplets causes the directors to reorient faster than the ellipse since they are mostly parallel to each other and have less elastic energy than the circle, as illustrated in Figure 5. It is the elastic distortion that causes free energy to rise, which drives a reorientation from a bipolar to radial orientation within the elongated droplet. Thus, lower aspect ratios lead to a faster decline in the mean magnitude of the angle.

Since the mean angle gradually decreases, a characteristic time has been defined as we used in our past papers [17,39,41]. To investigate the average orientation over time within the droplet, the data are normalized, and the time constant is defined as 36.78 percent of the declining process. It helps characterize how quickly the droplet reorients to the radial configuration.

**Figure 6:**
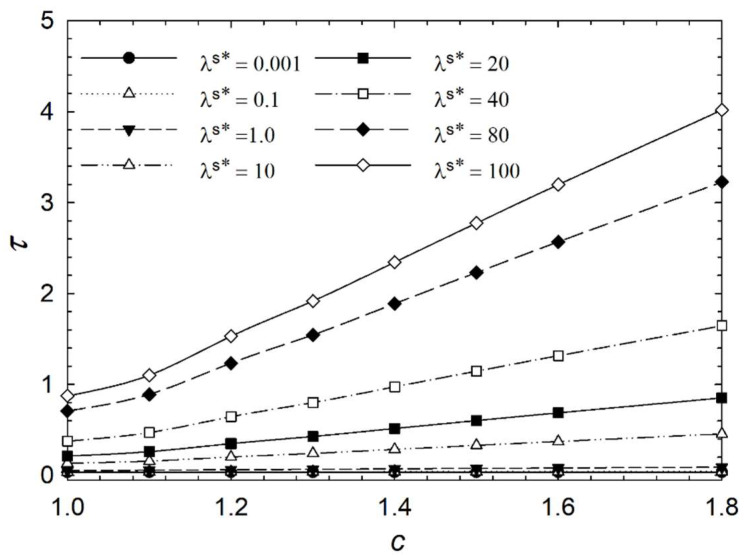
Characteristic time *τ* of the liquid crystal biosensor droplet versus aspect ratio *c* at dimensionless anchoring energy *W* = 10 at different dimensionless surface viscosities *λ*^s^*.

**Figure 7:**
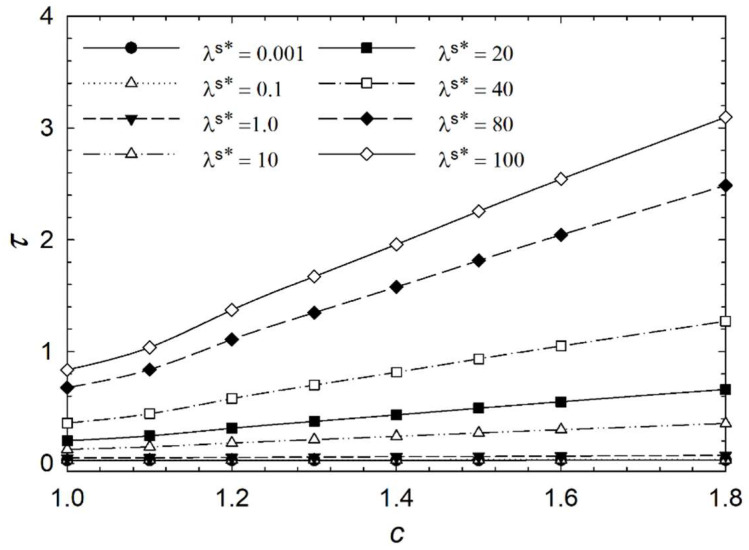
Characteristic time *τ* of the liquid crystal biosensor droplet versus aspect ratio *c* at dimensionless anchoring energy *W* = 20 at different dimensionless surface viscosities *λ*^s^*.

**Figure 8:**
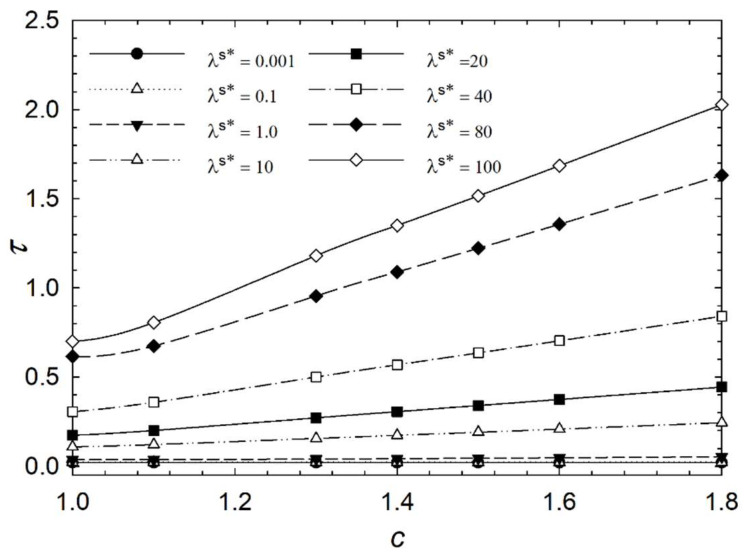
Characteristic time *τ* of the liquid crystal biosensor droplet versus aspect ratio *c* at dimensionless anchoring energy *W* = 50 at different dimensionless surface viscosities *λ*^s^*.

**Figure 9:**
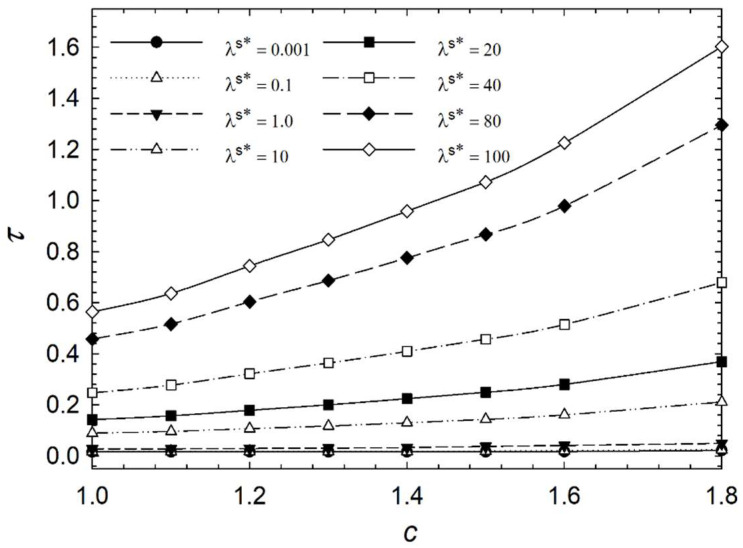
Characteristic time *τ* of the liquid crystal biosensor droplet versus aspect ratio *c* at dimensionless anchoring energy *W* = 100 at different dimensionless surface viscosities *λ*^s^*.

Figure 6, Figure 7, Figure 8 and Figure 9 show the characteristic time of the process in relation to the aspect ratio at different cases for homeotropic anchoring as follows: *W* = 10, *W* = 20, *W* = 50, and *W* = 100 with a range of 10^−3^ < λ^s^* ≤ 10^2^. Figure 6, Figure 7, Figure 8 and Figure 9 show how characteristic time τ varies with the aspect ratio in linear scale relation for various dimensionless surface viscosities. These figures show the upward trend of characteristic time with *c*. Also, it illustrates that the characteristic time increases with increasing surface viscosity. Based on the results, the characteristic time is influenced by the viscosity of the surface and the aspect ratio. Furthermore, it was found that the aspect ratio has less impact on response time for *λ*^s^* ≤ 1.0 for all studied cases with different homeotropic anchoring energies. The results suggest that the characteristic time is mainly affected by the viscosity of the surface. Moreover, the aspect ratio has a minimal influence on response time when the surface viscosity is low. Figure 6, Figure 7, Figure 8 and Figure 9 illustrate a reasonably consistent trend; however, by increasing homeotropic anchoring for 10 < *W* ≤ 100, the response time of the process decreases. In addition, at *W* = 100 it observes more gradual changes over time for *λ*^s^* ≥ 10.

**Figure 10:**
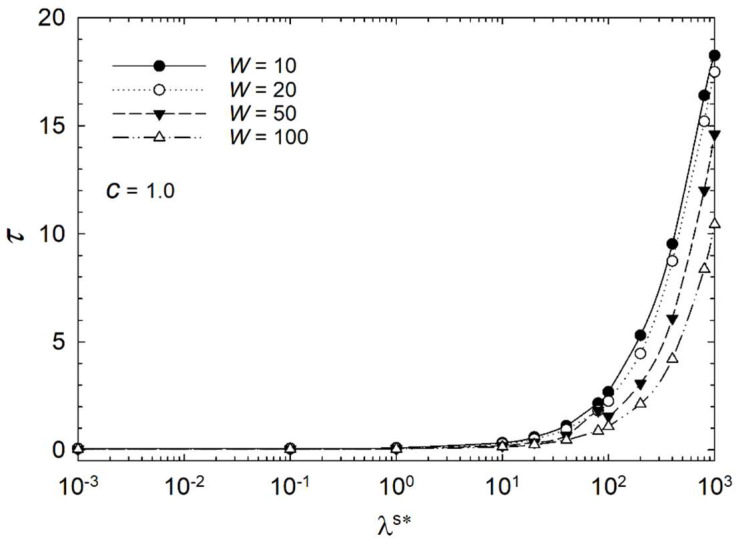
Characteristic time *τ* of the liquid crystal biosensor droplet versus dimensionless surface viscosity *λ*^s^* at aspect ratio *c* = 1.0 at various dimensionless anchoring energies.

**Figure 11:**
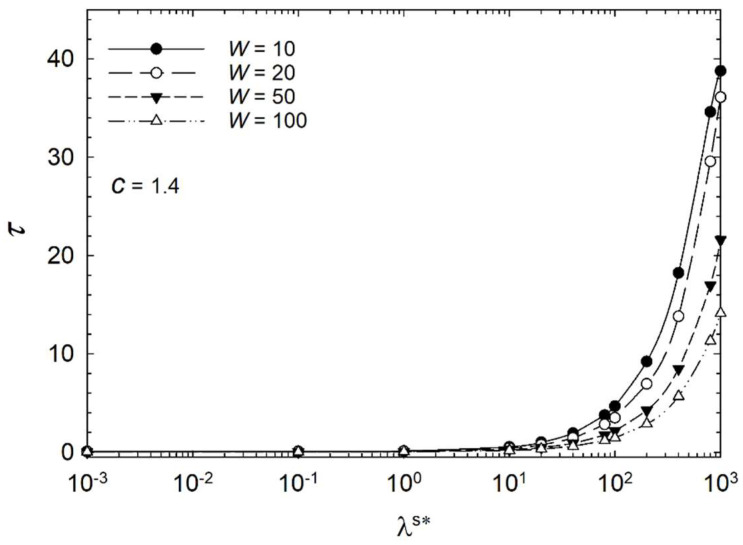
Characteristic time *τ* of the liquid crystal biosensor droplet versus dimensionless surface viscosity *λ*^s^* at aspect ratio *c* = 1.4 at various dimensionless anchoring energies.

**Figure 12:**
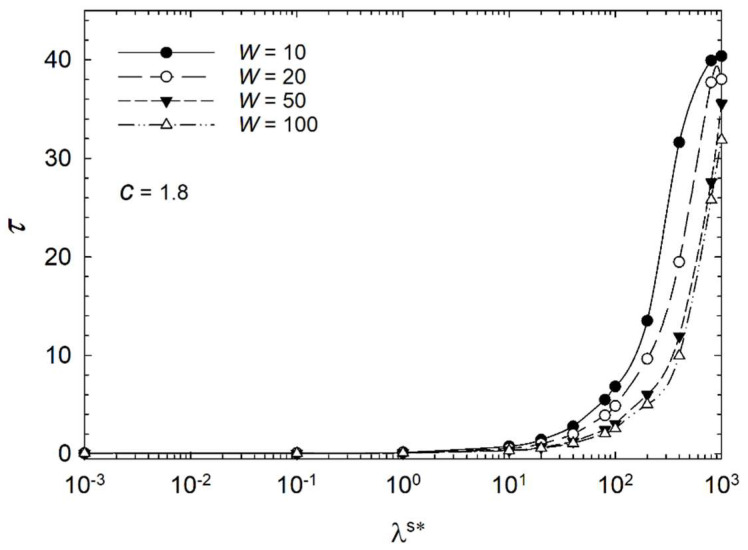
Characteristic time *τ* of the liquid crystal biosensor droplet versus dimensionless surface viscosity *λ*^s^* at aspect ratio *c* = 1.8 at various dimensionless anchoring energies.

For a better understanding of the influence of the surface parameters, Figure 10, Figure 11 and Figure 12 show semilogarithmic plots of characteristic time versus dimensionless surface viscosity λ^s^* at various homeotropic anchoring strengths in the range of 10^1^ < *W* < 10^2^ for three cases of aspect ratio *c* = 1.8, *c* = 1.4, and *c* = 1.0. The plots indicate that the characteristic time decreases with increasing surface viscosity for *λ*^s^* ≥ 10^1^ for all anchoring strengths. The strongest homeotropic anchoring strength shows the fastest trend. Furthermore, the stronger homeotropic anchoring *W* = 50 and *W* = 100 are almost overlapping in the case of *c* = 1.8.

As illustrated in Figure 10, Figure 11 and Figure 12, the relation between characteristic time and *W* is independent of the dimensionless surface viscosity in the range *λ*^s^* ≤ 1.0. Furthermore, the dark appearance of droplet interference in the steady state does not vary with surface viscosity within this range. However, the surface viscosity affects the transient process within the droplet and the biosensor performance for λ^s^* ≥ 10^0^. Thus, the decay time of the elongated bipolar droplet during the response to the surface interaction depends primarily on the dimensionless surface viscosity.

Therefore, the dimensionless surface viscosity *λ*^s^* and dimensionless anchoring energy *W* should also be taken into account when designing biosensors. The characteristic time for a distorted configuration to achieve radial orientation depends on these parameters. The aspect ratio also impacts the biosensor’s response time; however, the response time is not affected by the aspect ratio at the limit of low surface viscosity. At the limit of high surface viscosity, the response time is strongly affected by the aspect ratio. Thus, when designing biosensors, all three parameters should be considered.

**Figure 13:**
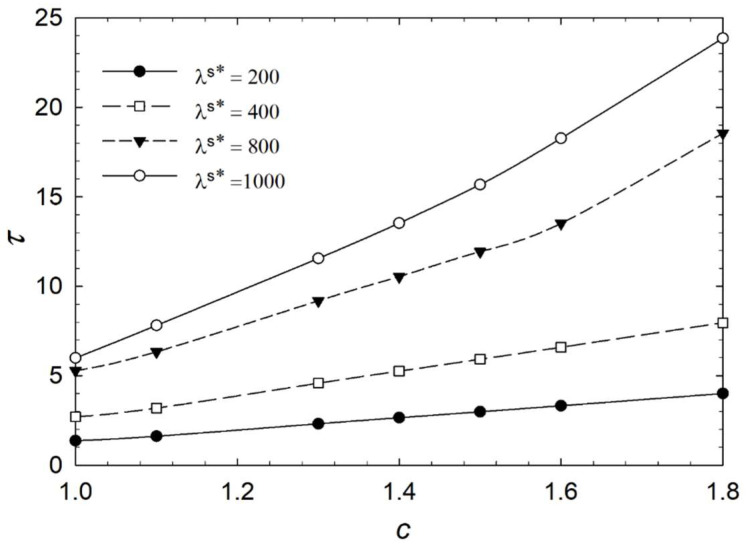
Characteristic time *τ* of the liquid crystal biosensor droplet versus aspect ratio *c* at dimensionless anchoring energy *W* = 50 at *λ*^s^* > 100.

**Figure 14:**
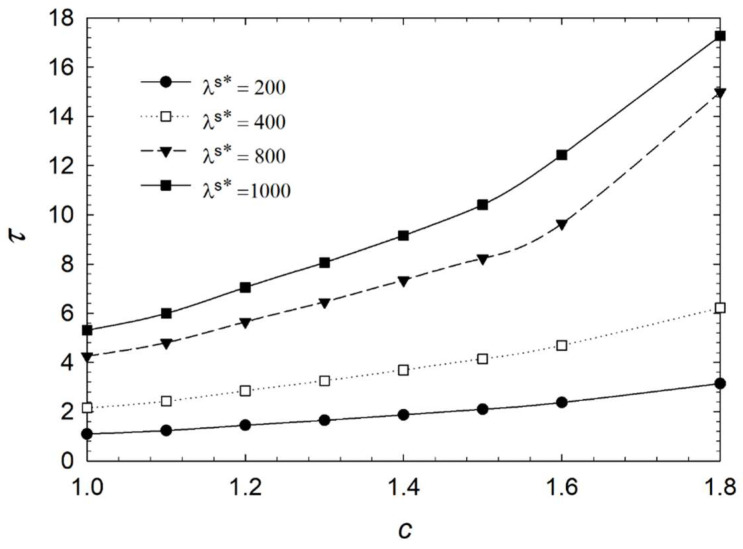
Characteristic time *τ* of the liquid crystal biosensor droplet versus aspect ratio *c* at dimensionless anchoring energy *W* = 100 at *λ*^s^* > 100.

Considering surface viscosity has two distinct trends in Figure 10, Figure 11 and Figure 12, Figure 13 and Figure 14 illustrate the characteristic time versus aspect ratio for a higher range of viscosity in the range of 2 × 10^2^ ≤ *λ*^s^* ≤ 10^3^. Although the dynamic is the same when viscosity is higher, it shows a higher upward trend of characteristic time with respect to aspect ratio than when viscosities are lower. It is recommended to keep the aspect ratio low when operating biosensors with higher dimensionless surface viscosity.

## 4. Conclusions

In this study, the effects of elongated droplet shape in relation to the surface properties were studied. Our study indicates that the director reorients faster in the range where the ellipse has a smaller aspect ratio. The results showed that the director reorientation dynamics are strongly dependent on the aspect ratio and surface viscosity, as well as on homeotropic anchoring energy.

We found that the reorientation of the liquid crystal molecules is affected by the shape of the droplet, and elongated droplets exhibit slower reorientation than spherical droplets. This suggests that the shape of the droplet can be used to control the performance of liquid crystal biosensors. The results of this study have implications for the design of liquid crystal biosensors, as surface viscosity is shown to be a key factor in controlling droplet orientation. Therefore, manipulating surface viscosity could lead to improved sensitivity and precision in droplet-based liquid crystal biosensors.

During the design of biosensors and droplet fabrication, it is recommended to consider low aspect ratios as a performance criterion for fast bulk responses to molecular interactions at droplet surfaces in the range of dimensionless λ^s^* ≥ 10 in order to achieve a desirable performance. According to the numerical results, spherical or slightly elongated droplets are suitable for biosensor applications. Also, the effect of the aspect ratio was found to be lower for low surface viscosity.

## Data Availability

Not applicable.

## Nomenclature

**Symbols Description**	
*A*	rate of strain tensor
*F*	total energy
*F*_s_	surface energy
*F*_d_	Frank elastic free energy density per volume
*h*	film thickness
**h**	the molecular field
**h**_s_	splay molecular field
**h**_T_	twist molecular field
**h**_B_	bend molecular field
*I*	the light intensity
*K*	elastic constant
*K*_1_	splay elastic constant
*K*_2_	twist elastic constant
*K*_3_	bend elastic constant
**n**	directo
**N**	co-rotational time flux
*R*	Rayleigh dissipation function
*t*	time
**V**	velocity
*V*	volume
*W*	anchoring energy
**Greek Symbols**	
**Γ**	torque per unit volume
**Γ**_e_	elastic torque
**Γ**_v_	viscous torque
*γ*_1_	rotational viscosity coefficient
*γ*_2_	irrotational torque coefficient
*η_i_*	angle-dependent viscosity functions
ϕ	polar angle—zenithal angle
*κ_i_*	angle-dependent elastic functions
*λ* ^s^	surface viscosities
*ν*	unit normal vector
τ	time constant
**Ω**	angular velocity tensor

## Appendix A

The elastic functions are defined as follows:

(A1) $$\kappa_{1}=\frac{1}{2}\left(\left(K_{11}^{*}-K_{33}^{*}\right)\cos\left(2\phi \right)+K_{11}^{*}+K_{33}^{*}\right)$$

(A2) $$\kappa_{2}=\frac{1}{2}\left(\left(K_{33}^{*}-K_{11}^{*}\right)\cos\left(2\phi \right)+K_{11}^{*}+K_{33}^{*}\right)$$

(A3) $$\kappa_{3}=\left(K_{33}^{*}-K_{11}^{*}\right)\sin\left(2\phi \right)$$

(A4) $$\kappa_{4}=\frac{1}{2}\left(K_{33}^{*}-K_{11}^{*}\right)\sin\left(2\phi \right)$$

(A5) $$\kappa_{5}=\frac{1}{2}\left(K_{11}^{*}-K_{33}^{*}\right)\sin\left(2\phi \right)$$

(A6) $$\kappa_{6}=\left(K_{33}^{*}-K_{11}^{*}\right)\cos\left(2\phi \right)$$

(A7) $$\kappa_{7}=\frac{1}{2}\frac{1}{r^{*}}\left(\left(K_{11}^{*}-K_{33}^{*}\right)\cos\left(2\phi \right)+K_{11}^{*}+K_{33}^{*}\right)$$

(A8) $$\kappa_{8}=\frac{1}{2}\frac{1}{r^{*}}\left(K_{33}^{*}-K_{11}^{*}\right)\sin\left(2\phi \right)$$

(A9) $$\kappa_{9}=-\frac{1}{2}\frac{1}{r^{*}{}^{2}}K_{11}^{*}\sin\left(2\phi \right)$$

(A10) $$\kappa_{10}=\frac{z^{*}}{r^{*}}K_{11}^{*}\sin^{2}\phi -\frac{1}{2}K_{11}^{*}\sin\left(2\phi \right)$$

(A11) $$\kappa_{11}=\frac{1}{2}\left(K_{11}^{*}-K_{33}^{*}\right)z^{*}\sin\left(2\phi \right)-r^{*}\left(K_{11}^{*}\cos^{2}\phi +K_{33}^{*}\sin^{2}\phi \right)$$

(A12) $$\kappa_{12}=\frac{1}{2}\left(K_{33}^{*}-K_{11}^{*}\right)r^{*}\sin\left(2\phi \right)-z^{*}\left(K_{11}^{*}\sin^{2}\phi +K_{33}^{*}\cos^{2}\phi \right)$$
